# Draft Genome Sequence of Pseudomonas sp. Strain MWU13-2860, Isolated from a Wild Cranberry Bog in Truro, Massachusetts

**DOI:** 10.1128/MRA.01007-18

**Published:** 2018-10-04

**Authors:** Ghazal Ebadzadsahrai, Jonathon Thomson, Scott Soby

**Affiliations:** a Biomedical Sciences Program, College of Graduate Studies, Midwestern University, Glendale, Arizona, USA; b College of Dental Medicine, Midwestern University, Glendale, Arizona, USA; c Biomedical Sciences Program, College of Graduate Studies and College of Veterinary Medicine, Midwestern University, Glendale, Arizona, USA; University of California, Riverside

## Abstract

Pseudomonas sp. strain MWU13-2860 was isolated from the rhizosphere of wild cranberry plants and is not closely related to Pseudomonas spp. frequently isolated from soil. Its genome is 7.2 Mbp, with 61.24% G+C content, and contains homologs that may encode the carbohydrate-degrading enzymes xylanase, laccase, cellulase, alginate lyase, amylase, and chitinase.

## Abstract (precis)

Pseudomonas sp. strain MWU13-2860 was isolated from the rhizosphere of wild cranberry plants and is not closely related to Pseudomonas spp.

## ANNOUNCEMENT

**FIG 1:**
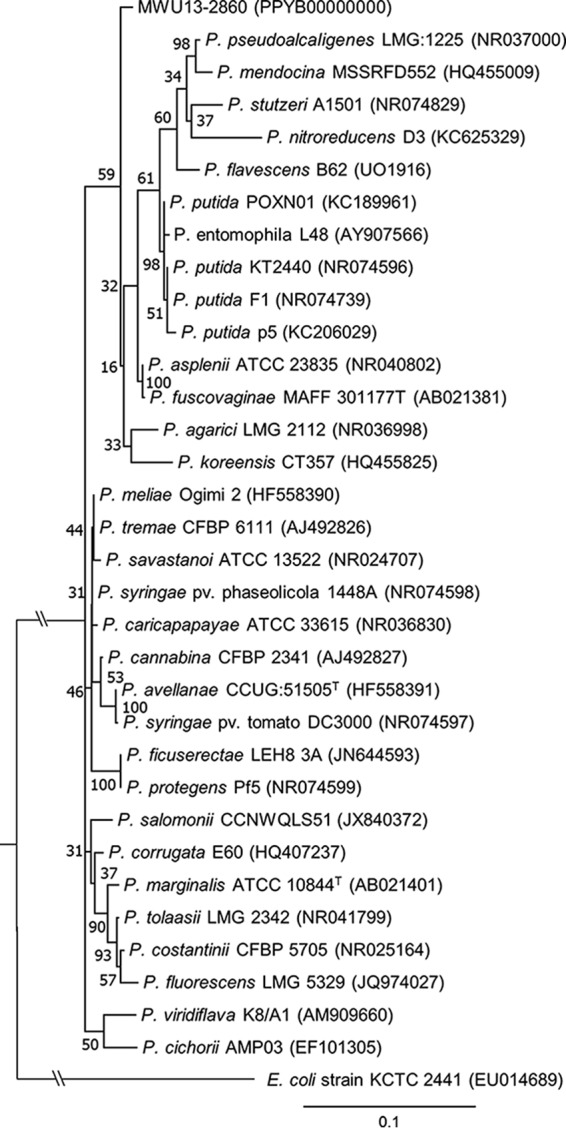
An evolutionary history (16S rRNA phylogeny) for Pseudomonas spp. commonly associated with soil and plant tissues, including isolate MWU13-2860, was inferred in MEGA7. Sequences were aligned by MUSCLE, and a maximum likelihood tree was constructed, with complete deletion of gaps and missing data, based on the Kimura 2-parameter model. The tree with the highest log likelihood (-4,713.97) is shown, with bootstrap values based on 500 iterations next to the branches. An initial tree was obtained by applying neighbor-joining and BioNJ algorithms to pairwise distances using the maximum composite likelihood (MCL) approach, followed by selecting the topology with a superior log-likelihood value. A discrete gamma distribution to model evolutionary rate differences among sites (+*G*, parameter = 0.1370) and a rate variation model that allowed for some sites to be evolutionarily invariable ([+*I*], 60.10% of the sites) were used. Except for the Escherichia coli outgroup, the tree is drawn to scale, with branch lengths measured in the number of substitutions per site. A total of 1,320 positions were used in the final data set.

Pseudomonas is a large and diverse genus whose members produce secondary metabolites (1) that influence the microbes and macrobiota, as well as the biogeochemical processes of soil (2–8). However, little information is available about which members of the genus are present or about the functional roles of Pseudomonas spp. in wetland bog soils. A number of previously uncharacterized Pseudomonas spp. were isolated from wild cranberry bogs at the Cape Cod National Seashore in Massachusetts as part of a culture-dependent survey of bog soil bacteria. Here, we report the draft genome sequence of Pseudomonas sp. strain MWU13-2860, an isolate that by 16S RNA phylogeny (9, 10) is apparently not closely related to other Pseudomonas spp. commonly isolated from soil or plant tissues (Fig. 1).

Wild cranberry bog soil and roots were seeded onto King’s medium B (KMB) agar supplemented with 50 μg ml^-1^ cycloheximide and ampicillin and grown at 26°C. Isolate MWU13-2860 was single-colony purified 3 times on KMB agar and grown overnight in KMB broth cultures for genomic DNA (gDNA) extraction (DNeasy blood and tissue kit; Qiagen). The genomic DNA of MWU13-2860 was sheared to approximately 600 bp by ultrasonication (Covaris M220), and libraries were generated on an Apollo 384 liquid handler (Wafergen) for Illumina sequencing using a library preparation kit (catalog number KK8201; Kapa Biosystems). DNA fragments were end repaired and A tailed before ligation with combination indexes/adapters (catalog number 520999; Bioo). Adapter-ligated DNA fragments were prepared for amplification with Kapa HiFi enzyme with AMPure beads (catalog number A63883; Agencourt Bioscience/Beckman Coulter, Inc.). The resultant libraries were assessed on an Agilent Bioanalyzer and by quantitative PCR (catalog number KK4835, library quantification kit; Kapa). Samples were then pooled and sequenced in 2 × 300- and 2 × 150-bp paired-end flow cells on the MiSeq platform. The 2 × 300- and 2 × 150-bp read files were combined, partially assembled, and annotated on the PATRIC Bacterial Bioinformatics Resource Center website (http://patricbrc.org) using the Comprehensive Genome Analysis Pipeline, with default parameters (11). The autoassembly function of PATRIC runs BayesHammer, followed by Velvet, IDBA, and SPAdes (12–15). The genomic sequence had a coverage of 126× and consisted of 7,205,080 bp on 51 contigs (61.24% G+C content). The *N*_50_ value is 307,730 bp, and the largest contig is 752,227 bp.

Annotation in the PATRIC pipeline uses RAST*tk* (16). Pseudomonas MWU13-2860 contained 8,978 protein-coding genes. The genome also contains 61 tRNA and 6 rRNA operons. Most strikingly, MWU13-2860 contains homologs for multiple putative polysaccharide-degrading genes, including those for xylanases, laccases, and chitinases, as well as the hydrolytic exoenzymes cellulase, alginate lyases, and amylases. These putative genes suggest that this microorganism plays a role in the turnover of complex carbohydrates in the rhizosphere and soil of wetland bogs. The genome of this bacterium also possesses a number of potential virulence factor genes, including those for several proteinase inhibitors, type II and III secretion systems, and type VI secretion system lipoprotein.

### Data availability.

This whole-genome shotgun project has been deposited at DDBJ/EMBL/GenBank under the accession number PPYB00000000 for Pseudomonas MWU13-2860. The version described in this paper is PPYB02000000. The Sequence Read Archive (SRA) accession number is SRX4454450.
